# Investigation of the Molecular Mechanism of Asthma in Meishan Pigs Using Multi-Omics Analysis

**DOI:** 10.3390/ani15020200

**Published:** 2025-01-13

**Authors:** Weilong Tu, Hongyang Wang, Yingying Zhang, Ji Huang, Yuduan Diao, Jieke Zhou, Yongsong Tan, Xin Li

**Affiliations:** 1 Institute of Animal Husbandry and Veterinary Science, Shanghai Academy of Agricultural Sciences, Shanghai 201106, China; tuweilong@saas.sh.cn (W.T.); wanghongyang@saas.sh.cn (H.W.); zhangyingying@saas.sh.cn (Y.Z.); android717@gmail.com (J.H.); 20220603@saas.sh.cn (Y.D.); zhoujieke@saas.sh.cn (J.Z.); 2 Key Laboratory of Livestock and Poultry Resources (Pig) Evaluation and Utilization, Ministry of Agriculture and Rural Affairs, Shanghai 201106, China; 3 Shanghai Engineering Research Center of Breeding Pig, Shanghai 201106, China

**Keywords:** Meishan pig, asthma, transcriptome, metabolome

## Abstract

Asthma has been extensively studied in humans and animals, but the molecular mechanisms underlying asthma in Meishan pigs, a breed with distinct genetic and physiological characteristics, remain elusive. Understanding these mechanisms could provide insights into veterinary medicine and human asthma research. We investigated asthma pathogenesis in Meishan pigs through transcriptomic and metabolomic analyses of blood samples taken during autumn and winter. Asthma in Meishan pigs is related to inflammation, mitochondrial oxidative phosphorylation, and tricarboxylic acid (TCA) cycle disorders. Related genes include *CXCL10*, *CCL8*, *CCL22*, *CCL21*, *OLR1*, and *ACKR1*, while metabolites include succinic acid, riboflavin-5-phosphate, and fumaric acid. Transcriptomic sequencing was performed on panting and normal Meishan pigs, and differentially expressed genes underwent functional enrichment screening. Metabolomic analysis revealed differential metabolites and pathways between groups. Combined analyses indicated that lung inflammation is influenced by genetic, allergenic, and environmental factors disrupting oxidative phosphorylation in lung mitochondria, affecting the TCA cycle. Mitochondrial reactive oxygen species, glutathione S-transferases, arginase 1 and RORC in immune regulation, the Notch pathway, YPEL4 in cell proliferation, and MARCKS in airway mucus secretion play roles in asthma pathogenesis. This study highlights that many cytokines and signaling pathways contribute to asthma. Further studies are needed to elucidate their complex interactions.

## Simple Summary

Asthma is a common respiratory disorder; however, its molecular mechanisms in Meishan pigs are not well understood. This study aimed to investigate using transcriptomic and metabolomic analyses of blood samples, genes like *CXCL10* and metabolites such as succinic acid were found to be related to asthma. Results suggest asthma is influenced by genetic, allergenic, and environmental factors. The study improves our understanding of this disease in Meishan pigs, potentially aiding the breeding of resistant pigs and providing insights into veterinary medicine and potentially also human asthma research due to the physiological similarities between pigs and humans.

## 1. Introduction

Asthma is a highly prevalent chronic respiratory disease that affects up to 358 million people worldwide [1]. Eighty percent of individuals with asthma receive treatment; however, 50% of cases are not well controlled. According to an epidemiological survey in China, the prevalence of asthma in people aged 20 years and older was 4.2%; only 28.5% of patients with asthma had well-controlled disease, while 70–80% of asthma were uncontrolled [2]. Treatment costs for patients with uncontrolled asthma are twice those of patients with controlled asthma. Similarly, pigs with asthma show chronic stunting, with production efficiency and feed utilization decreasing by 15.9% and 13.8%, respectively [3]. The Meishan pig, a Taihu pig breed in Southern China known for its high fertility and good meat quality, is an important animal model [4]. In the Jiading District, asthma has been a persistent problem in Meishan pig breeding. Despite a relatively low mortality rate from asthma, it hampers the growth rate of Meishan pigs, creating significant difficulties in breeding and resulting in substantial economic losses for pig farms. Therefore, understanding the pathogenesis of asthma in Meishan pigs is crucial for both veterinary medicine and potentially human asthma research, as pigs share many physiological similarities with humans.

Asthma manifests as inflammation and hyper-responsiveness of the airways. Asthma has substantial impacts at both clinical and mechanistic levels [5]. Inflammation is the core of asthma pathogenesis, and the main cause of airflow obstruction and airway hyperreactivity [6]. An imbalance between T helper type 1 (Th1)/T helper Type 2 (Th2) cells has been highlighted in the pathogenesis of asthma [7], which is characterized by the predominance of Th2 cells. Analysis of metabolites in urine from asthmatic and healthy individuals revealed that alterations in the citric acid cycle (tricarboxylic acid [TCA] cycle) appeared to be the main differences between individuals with and without asthma [8]. Although research has accelerated, and substantial advances in asthma treatment have been made, the standard treatment approach is largely based on immunosuppressants and bronchodilators, which provide short-term relief, but are not curative. Existing treatment methods cannot fully meet clinical needs; therefore, current research is focusing on the search for new and effective treatment methods for asthma.

Our team found that asthma attacks in Shanghai Jiading Meishan pigs are seasonal, occurring especially often in autumn and winter, or winter and spring. Therefore, developing asthma-resistant pigs is a vital research direction for Meishan pig breeding. Thus, we analyzed the blood of panting and healthy Meishan pigs using transcriptomics and metabolomics to ascertain the molecular mechanisms underlying asthma attacks in Meishan pigs, providing a reference for breeding asthma-resistant Meishan pigs.

## 2. Materials and Methods

### 2.1. Sample Preparation

The experiment was conducted from June to December 2020, and Meishan pigs were bred at the Meishan Pig Breeding Center, Jiading District, Shanghai. Throughout the experiment, all Meishan pigs were raised under completely identical and highly controlled environmental conditions. They were housed in a specially designed facility with the temperature maintained at 22 ± 2 °C and the humidity kept at 50–60%. The pigs had free access to clean drinking water, which was sourced from the local municipal water supply and further purified through a reverse osmosis filtration system to ensure its quality. In terms of feeding, a standardized commercial pig feed (feed brand name: [Heima Feed]; manufacturer: [Shanghai Heima Feed Co., Ltd.; Shanghai; China]) was used. The feed formulation was designed to meet the specific nutritional requirements of Meishan pigs at their growth stage and consisted of a balanced combination of grains (such as corn and wheat), protein sources (including soybean meal and fish meal), essential vitamins (such as vitamins A, D, and E), and minerals (such as calcium, phosphorus, and iron). The feeding method was consistent, with the feed provided three times a day at 08:00, 12:00, and 17:00. Owing to the transition between autumn and winter seasons, the temperature changed abruptly, resulting in some pigs panting. The assessment was conducted based on clinical symptoms and auscultation. Specifically, pigs with asthma coughed relatively frequently, which could be a dry cough or a wet cough with a small amount of mucus. When using a stethoscope to auscultate the lungs of Meishan pigs, wheezing sounds might be heard in the lungs of asthmatic pigs. This is a high-pitched and continuous breathing sound caused by the airway narrowing and restricted airflow and is similar to a whistle or a crowing sound. In contrast, healthy pigs rarely coughed. Occasionally, they might cough slightly due to environmental stimuli, etc., but the cough was clear, short, and infrequent. When auscultated with a stethoscope, the breathing sounds in the lungs of healthy pigs were clear, soft, and without any murmurs or abnormal sounds. Eleven Meishan pigs with obvious asthma symptoms were randomly selected from a group of pigs with similar body types and labeled TWL1, TWL2, TWL3, TWL4, TWL7, TWL9, TWL10, TWL11, TWL12, TWL14, and TWL15. Eleven healthy Meishan pigs were included, which were labeled TWL17, TWL18, TWL19, TWL20, TWL21, TWL23, TWL24, TWL25, TWL27, TWL28, and TWL29.

To ensure that the pigs did not move during the operation, binding devices, such as ropes and specially made fixing frames, were used to fix the limbs and bodies of the pigs to an operating table. The sampling method of lung tissue was referred to the method reported by predecessors [9]. The anesthesia of the experimental pigs was achieved through intramuscular injection of a combination of ketamine hydrochloride and xylazine hydrochloride. The dosage of ketamine hydrochloride was 10–15 mg/kg of body weight, and the dosage of xylazine hydrochloride was 1–2 mg/kg of body weight. This combination of anesthetic drugs was chosen because it provides effective anesthesia with relatively few side effects and a suitable duration of action for the sampling procedure. After the pigs were anesthetized, lung tissue and blood samples were taken. The surgical area was thoroughly disinfected using Iodophor, which was wiped from the center of the surgical area to the periphery. A scalpel was used to make a 10–15 cm incision on the right side of the chest. After cutting through the skin, the subcutaneous tissue and muscle layers were carefully cut open with surgical scissors, taking care to avoid damaging deep blood vessels and nerves. After cutting through the muscle layer, the fascia and other soft tissues in the chest cavity were separated with anatomical forceps and surgical scissors, and the section between the 5th and 7th ribs was removed with a bone saw. Next, a retractor was used to open the chest cavity incision and fully expose the right lung. After exposing the right lung, about 1–2 cubic centimeters of lung tissue was carefully grasped with anatomical forceps, taking care to maintain the integrity of the tissue as much as possible, avoiding excessive squeezing or tearing. The collected lung tissue was quickly placed in a pre-prepared 4% paraformaldehyde solution for fixation, taking care that the solution sufficiently covered the tissue to ensure the tissue was fully fixed. After the lung tissue was collected, the surgical wound was treated. After the operation, the pigs were closely observed to ensure good recovery, and appropriate care and treatment were given, such as using antibiotics to prevent infection. Right lung tissue was collected and fixed in 4% paraformaldehyde. The fixed lung tissue was dehydrated, paraffin-embedded, stained with hematoxylin and eosin (H&E) according to previously described methods [10], and photographed for observation.

The Meishan pigs were restrained on a frame to ensure that they would not struggle violently during the blood collection process. The sampling method of the blood sample was referred to the method reported by predecessors [11]. The approximate location of the anterior vena cava was found by touching the neck of the pig at the depression above the manubrium of the sternum. The blood collection site was wiped and disinfected with an iodine-soaked cotton ball, wiping an area of 5–10 cm in diameter from the center of the site. Then, an alcohol-soaked cotton ball was used to disinfect the site. A tourniquet was used to moderately compress the anterior vena cava above the blood collection site (towards the head), ensuring the vein was full and facilitating the accurate insertion of the blood collection needle. The needle was quickly inserted into the anterior vena cava at a 30–45° angle to the skin surface. After collection, the tourniquet was quickly released, and the needle was removed. A disinfected cotton ball was used to press on the blood collection site for a moment to prevent bleeding. The samples were labeled and stored for subsequent use.

### 2.2. Transcriptome Sequencing Analysis

#### 2.2.1. cDNA Library Construction and Transcriptome Sequencing

More than 90% of the RNA in the blood samples of Meishan pigs is rRNA; therefore, after extracting total RNA from the samples, rRNA was removed using conventional kits and mRNA enriched. The enriched mRNA was reverse-transcribed to form double-stranded cDNA. After repairing the double ends of the cDNA, a machine library was constructed by PCR amplification with splices. An Illumina Truseq^TM^ RNA sample prep kit was used to construct the library. To ensure sequencing data quality, the following quality checks were performed.

Agarose gel electrophoresis (1.5%): Assessed RNA integrity of the sample and checked for DNA contamination. The electrophoresis was performed using a Bio-Rad electrophoresis system (Bio-Rad Laboratories, Hercules, CA, USA) according to previous reports [12]. NanoPhotometer spectrophotometry: Measured RNA purity (OD_260/280_ and OD_260/230_ ratio). The NanoPhotometer used was from Implen GmbH (Munich, Germany), and the operation was carried out based on a previously reported method [13]. Qubit2.0 Fluorometer: Precisely quantified RNA concentration. The Qubit2.0 Fluorometer is a product of Thermo Fisher Scientific (Waltham, MA, USA), and the quantification was performed using the Qubit RNA HS Assay Kit following the provided protocol [14]. Agilent 2100 bioanalyzer: Examined RNA integrity. The Agilent 2100 bioanalyzer was used with the Agilent RNA 6000 Nano Kit according to the standard operating procedures of Agilent Technologies (Santa Clara, CA, USA) [15].

Once the quality of the constructed library was confirmed, second-generation sequencing was performed by pooling different libraries according to the requirements of effective concentration and target data volume. Sequencing was conducted using a sequencing-by-synthesis approach.

#### 2.2.2. Sequence Alignment

Raw sequencing reads were filtered to remove low-quality base sequences, such as splices and poly-N stretches, generating a set of high-quality clean reads for further analysis. Quality control was performed using Trimmomatic 0.40 (http://www.usadellab.org/cms/index.php?page=trimmomatic, accessed on 1 November 2024) with the following filtering steps:

(1) The joint sequences in reads were removed and reads without insert fragments were removed.
(2) Low-quality bases (phred < 20) at the 3′ end were pruned. If any base had a mass value < 10, the entire sequence was eliminated; otherwise, it was retained.
(3) Reads containing more than 10% N were removed.
(4) Reads shorter than 75 bp in length after adapter removal and mass pruning were discarded.

The clean reads obtained were compared with a pig reference genome (Sus_scrofa.Sscrofa11.1) using Hisat2 (v2.0.5) software, which was run with default parameter setting [16].

#### 2.2.3. Calculation of Gene Expression and Differential Gene Screening

Read counts of each transcript were determined based on their locations in the genome, and the fragments of kilobase transcript per million mapped reads (FPKM) value was used to quantify gene expression levels. The amount of mRNA transcribed by each gene is regulated by multiple factors, such as time and space, and the amount of mRNA transcribed by genes varies at different growth and development stages or at different tissue levels in Meishan pigs.

$$\mathrm{FPKM}=\frac{\mathrm{total}\;\mathrm{exon}\;\mathrm{Fragments}}{\mathrm{mappedreads}\left(\mathrm{millions}\right)\times \mathrm{exon}\;\mathrm{length}\;(KB)}$$

Higher FPKM values indicate higher gene expression levels. Differentially expressed genes (DEGs) were screened by setting a threshold value for the false discovery rate (FDR). In the comparison between the two groups, the screening criteria for DEGs were |log_2_ fold change(FC)| > 0 and FDR < 0.05. The statistical analysis was performed using the DESeq2 (v1.30.0) software (https://bioconductor.org/packages/release/bioc/html/DESeq2.html, accessed on 10 November 2024), which is a widely used tool for differential gene expression analysis in RNA-seq data [17].

### 2.3. Metabolome Determination and Analysis

#### 2.3.1. Extraction of Metabolites

We performed non-targeted metabolomics using liquid chromatography–mass spectrometry (LC-MS). Blood samples were collected with a green cap heparin anticoagulant tube, after which the plasma was separated as soon as possible, centrifuged at 3000 r for 10 min, and the upper layer of the subpackaged plasma was added to a 1.5 mL centrifuge tube. The 100 μL sample was placed in an EP tube and 400 μL of 80% methanol aqueous solution was added. The solution was vortexed, then placed on an ice bath for 5 min and centrifuged at 15,000× *g* and 4 °C for 20 min. The supernatant was then diluted with water until the methanol content reached 53%. It was then centrifuged at 4 °C at 15,000× *g* for 20 min and the supernatant was collected and injected into the LC-MS instrument for analysis. Equal-volume samples were taken from each experimental sample and mixed as QC samples to correct for deviations in the mixed sample analysis results and for errors caused by the analysis instrument itself. The remaining samples were used for LC-MS detection. The metabolite extraction protocol was adapted from a previously published method with some modifications [18].

#### 2.3.2. LC-MS Analysis

The experiments were conducted using a Hyperil Gold column (C18) column. Gradient elution was performed at a flow rate of 0.2 mL·min^−1^ with a column temperature of 40 °C with the 2 μL sample. Under normal mode, mobile phase A was 0.1% formic acid and mobile phase B was methanol. In the negative mode, the mobile phase A was 5 mmol·L^−1^ ammonium acetate, while the mobile phase B was methanol. Mass spectrometry was performed using a Thermo Fisher Scientific (Q Exactive^TM^ HF-X) mass spectrometer. Positive and negative ion detection was performed in the ESI mode, with a mass scanning range of 100–1500 *m/z*. The LC-MS analysis was carried out following the standard operating procedures of the instrument and the recommended methods for metabolomics analysis [19].

#### 2.3.3. Mass Spectrum Data Analysis

The raw data files generated by UHPLC-MS/MS were processed using Compound Discoverer 3.1 (CD3.1, Thermo Fisher Scientific) to perform peak alignment, peak picking, and quantitation for each metabolite. The main parameters were set as follows: retention time tolerance, 0.2 min; actual mass tolerance, 5 ppm; signal intensity tolerance, 30%; signal/noise ratio, 3; and minimum intensity, 100,000. Peak intensities were normalized to the total spectral intensity. Normalized data were used to predict the molecular formula based on the additive ions, molecular ion peaks, and fragment ions. The peaks were then matched using the mzCloud^TM^ (https://www.mzcloud.org/, accessed on 11 November 2024), mzVault, and MassList databases to obtain accurate qualitative and quantitative results. Statistical analyses were performed using R (version R-3.4.3) (https://www.r-project.org/, accessed on 12 November 2024) and Python (Python 2.7.6 version) (https://www.python.org/, accessed on 13 November 2024) on a CentOS server (CentOS release 6.6) (https://www.centos.org/, accessed on 14 November 2024). When data were not normally distributed, normal transformations were attempted using the area normalization method.

Metabolites were annotated using the Kyoto Encyclopedia of Genes and Genomes (KEGG) (https://www.genome.jp/kegg/pathway.html, accessed on 15 November 2024), HMDBdatabase (https://hmdb.ca/metabolites, accessed on 15 November 2024), and LIPID Maps (http://www.lipidmaps.org/, accessed on 15 November 2024) databases. Principal component analysis (PCA) and partial least squares discriminant analysis (PLS-DA) were performed using metaX (flexible and comprehensive software for processing metabolomic data) (https://github.com/changwn/metaX, accessed on 16 November 2024). We applied univariate analysis (*t*-test) to calculate the statistical significance. Metabolites with VIP > 1 and *p* < 0.05 and FC ≥ 2 or FC ≤ 0.5 were considered to be differential metabolites. Volcano plots were used to filter metabolites of interest based on the |log_2_FC| and log_10_(*p*-value) of the metabolites.

### 2.4. Graphic Production

For clustering heat maps, the data were normalized using z-scores of the intensity areas of differential metabolites and plotted using the Pheatmap package in R. Correlations between differential metabolites were analyzed using cor () in the R language (method = Pearson). Statistically significant correlations between differential metabolites were calculated using the cor.mtest () in R. *p* < 0.05 was set as the cut-off for statistical significance and correlation plots were constructed using the corrplot package in R. The functions of these metabolites and metabolic pathways were analyzed using the KEGG database. The metabolic pathway enrichment of differential metabolites was performed; when ratios were satisfied by x/n > y/N, the metabolic pathway was considered enriched. When the *p*-value of the metabolic pathway was <0.05, the metabolic pathway was considered statistically significantly enriched.

### 2.5. RT-qPCR to Verify the RNA-Seq Results

The total RNA of each sample was extracted according to the steps in the Trizol instruction manual, its concentration and purity were detected, and the qualified RNA was reverse-transcribed to synthesize cDNA.

#### 2.5.1. Primer Information for Selected Genes Used in qPCR Verification

For the purpose of validating the RNA—seq results through qPCR, six genes were randomly chosen (see Table 1). The primer sequences for these genes and the reference gene 18S are presented in the following table. These primers play a crucial role in accurately detecting the gene expression levels during the qPCR process.

#### 2.5.2. Genomic DNA Removal Reaction

Before proceeding with the subsequent experiments, it is necessary to remove the genomic DNA from the samples to ensure the accuracy of the experimental results. The specific conditions for the genomic DNA removal reaction are as Table 2.

#### 2.5.3. Retrotranscription Reaction

After the genomic DNA removal, the reverse transcription reaction needs to be carried out to synthesize cDNA. The reagents and their dosages for this process are as Table 3.

#### 2.5.4. qPCR Reaction System

To quantitatively analyze the target genes, the qPCR technique is employed. The reagents and their dosages in the qPCR reaction system are as Table 4.

#### 2.5.5. qPCR Reaction Parameters

The qPCR reactions of different genes follow specific parameter settings, which are crucial for accurately detecting the gene expression levels. The details are as Table 5.

The qPCR reaction was performed using a LightCycler 480 II Real-Time PCR System (Roche Diagnostics, Mannheim, Germany). The reaction mixture was prepared according to the manufacturer’s instructions for the SYBR Green PCR Master Mix (Takara Bio, Shiga, Japan) [20]. The cycling conditions were optimized based on the melting temperature of the primers and the characteristics of the target genes. The amplification efficiency of each primer pair was determined by generating a standard curve using a serial dilution of a pooled cDNA sample. The relative expression levels of the genes were calculated using the 2^−ΔΔCt^ method, with 18S as the internal reference gene [21].

### 2.6. Differentially Expressed Genes and Metabolite Enrichment Analysis

DEGs were analyzed using DESeq2 (v1.30.0). *p* < 0.05, and |FC| > 2 were used as the screening criteria. DEGs analyzed using topGO 3.20 software (Gene Ontology). Pathway significance enrichment analysis of KEGG was performed using ClusterProfiler software v3.18.1.

### 2.7. H&E Staining

Hematoxylin and eosin staining (H&E staining) is a fundamental technique in histology. Our assay was adapted from a protocol reported in a previous study [22]. Tissue samples were fixed with 10% buffered formalin. The fixed tissue samples were then sliced at approximately 4–5 microns. Then, the nuclei were stained with hematoxylin solution for 20–25 min and observed under a microscope until the nuclei exhibited a blue color. Subsequently, the cytoplasm was stained with eosin solution for approximately 10 s. Following staining, the tissue sections were dehydrated and made transparent by passing them through a series of alcohol concentrations and xylene. Finally, the section was sealed with a sealing tablet and was then ready for microscopic observation.

## 3. Result

### 3.1. Histology of Asthmatic Meishan Pig Lungs

**Figure 1:**
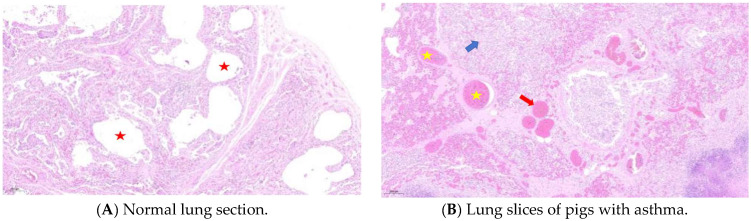
H&E staining of lung tissue. Compared with the normal group (**A**), lung sections from the panting group (**B**) showed alveolar cavity fusion, diffuse inflammatory lesions, small focal emphysema changes, parenchymal hemorrhage, interstitial microvascular dilation, neutrophil sequestration, and microthrombus formation. The red five-pointed star represents the alveolar cavity, the yellow five-pointed star represents alveolar cavity congestion, the red arrow represents the microthrombus, and the blue arrow represents diffuse inflammatory lesions.

Compared with healthy Meishan pigs, the pulmonary bronchioles of asthmatic pigs showed increased mucus, infiltration of blood vessels, and peribronchiolitis cells (Figure 1). In H&E staining images of healthy lung tissue, the cellular structure is well organized and neatly arranged. The alveolar epithelial cells and bronchial epithelial cells exhibit normal morphology with clear boundaries. There are few or almost no inflammatory cells present, indicating the absence of active inflammation. The airways are clear and unobstructed, with smooth muscle layers of appropriate thickness. The alveoli are intact and evenly distributed, facilitating efficient gas exchange. In contrast, images from asthmatic samples may show disruption of the overall structure. Epithelial cells may show signs of damage like swelling and irregular shapes. There is a significant increase in inflammatory cells such as eosinophils, neutrophils, and lymphocytes, which infiltrate the airway walls, alveolar septa, and surrounding tissues, indicating an active inflammatory response. The airways may be narrowed due to smooth muscle contraction and thickening of the airway wall. There may also be mucus plugging in the airways, further obstructing the flow of air. The alveoli may show signs of congestion and damage, with reduced gas exchange capacity. Additionally, in asthmatic lungs, there is often thickening of the submucosa due to increased deposition of extracellular matrix proteins and edema, further contributing to airway narrowing and impairment of lung function.

### 3.2. Transcriptomic Analysis Results

#### 3.2.1. Transcriptomic Sequencing Data of Panting and Normal Meishan Pigs

Transcriptomic sequencing was performed on blood samples from panting and normal Meishan pigs. A total of 1,489,104,280 raw and 1,470,084,608 clean reads were obtained from 11 samples in the asthma group. In total, 1,342,001,306 raw and 1,325,810,022 clean reads were obtained from the 11 healthy group samples (Table 6). After quality control filtering, 1,470,084,608 and 1,325,810,022 raw and clean reads were obtained in the two groups, respectively; the Q30 data of both groups were >92%, and the GC content was 53–58%. Comparison with the reference genome showed that the total comparison rate was >90% in both the healthy and panting groups. The data obtained by sequencing are reliable in quality and reliability and can be used for subsequent analyses. The relevant information is presented in Table 1.

#### 3.2.2. Cluster Analysis

**Figure 2:**
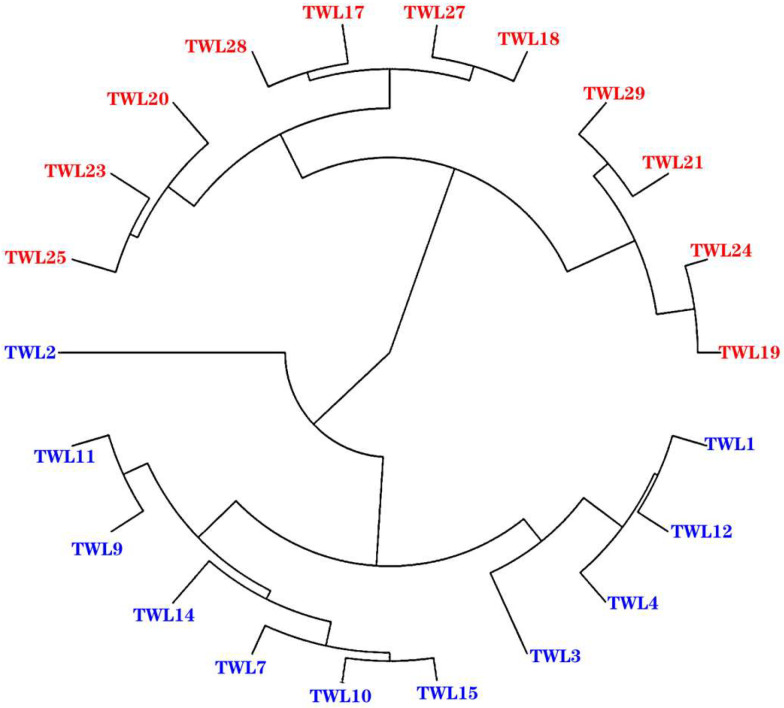
Manhattan map of the sample. The clustering method used was “average” and the distance calculation method was “manhattan”. Blue is the asthma group; red is the healthy group.

The samples were systematically clustered based on the expression of all the genes. As shown in Figure 2, asthma group samples were clustered together. Likewise, healthy group samples formed their own cluster. This pattern indicates that sampling was not incidental, but repeatable.

#### 3.2.3. Quantitative Real-Time RT PCR Validation of RNA-Seq Results

**Figure 3:**
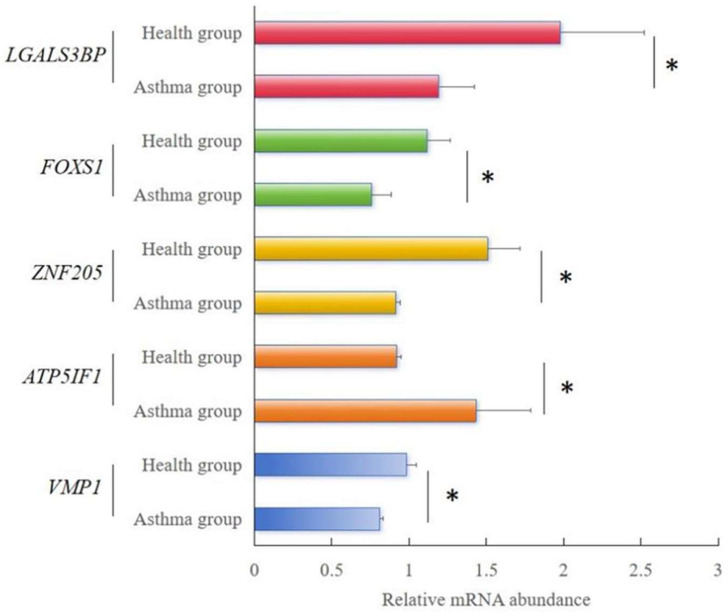

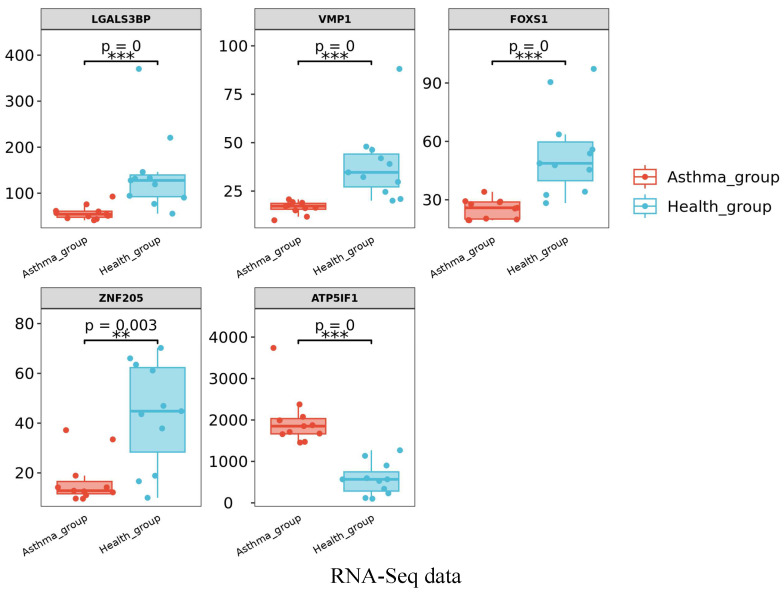
RT-qPCR analysis results of the relative mRNA abundance of five genes in Meishan pigs. The horizontal axis represents the type of sample, and the vertical axis represents the relative mRNA abundance normalized to that of β-actin. The relative mRNA abundance of 5 genes was represented as the mean, with error bars indicating standard error of the mean (mean ± SEM). Student’s *t*-test was used to assess the statistical significance of the experimental data (* *p* < 0.05; ** *p* < 0.01; *** *p* < 0.001). The results of RNA-seq showed the FPKM values of the five genes in different samples.

The relative expression of the five randomly selected genes was consistent with the variations observed in the RNA-seq analysis (Figure 3). Among these genes, *VMP1*, *ZNF205*, *ZNF205*, and *LGALS3BP* showed significant reductions (*p* < 0.05) in expression between the healthy and asthma groups. The relative expression levels of *LGALS3BP* were significantly increased (*p* < 0.05) between the healthy and asthma groups. Our qPCR results validated our RNA-Seq results, indicating that the results of Meishan pig transcriptomic sequencing are reliable and that subsequent experimental studies could be carried out.

#### 3.2.4. Analysis of Gene Expression Differences

**Figure 4:**
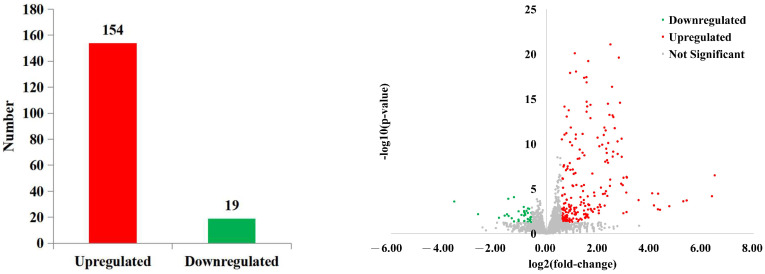
Differential expression analysis of genes.

In total, 173 DEGs were screened, of which 154 were upregulated and 19 were downregulated in the panting group (Figure 4).

#### 3.2.5. Functional Enrichment Analysis of Differentially Expressed Genes

**Figure 5:**
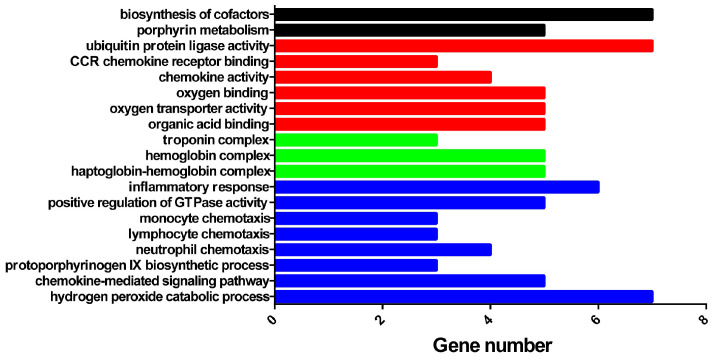
Enrichment analysis of differential genes.

After pathway enrichment, the main molecular function processes were found to be ubiquitin protein ligase activity, CCR chemokine receptor binding, chemokine activity, oxygen transporter activity, and organic acid binding (Figure 5). The main cellular component process terms were associated with organic acid binding and hemoglobin complexes. For biological processes, the key terms involved inflammatory responses, positive regulation of GTPase activity, monocyte chemotaxis, lymphocyte chemotaxis, neutrophil chemotaxis, protoporphyrinogen IX biosynthesis, chemokine-mediated signaling, and hydrogen peroxide catabolism. According to the KEGG metabolic pathway analysis, two pathways were identified, porphyrin metabolism and biosynthesis of cofactors.

### 3.3. Metabolome Determination and Analysis Results

#### 3.3.1. Metabolomic Database Annotation and Multivariate Statistical Analysis

**Figure 6:**
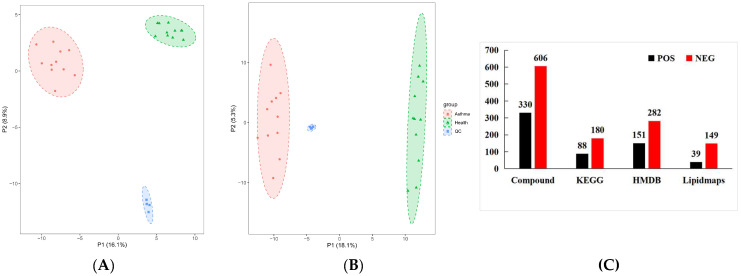
Statistical analysis of metabolomics data. Type, ion type of metabolite detection. Compound, the total number of metabolites identified under this ion type. KEGG, the number of metabolites annotated to the KEGG database. HMDB, the number of metabolites annotated to the HMDB database. Lipidmaps, the number of metabolites annotated to the LIPIDMAPS database. In the upper right corner of the graph, there is a legend that uses different colors and shapes to identify three groups. Among them, the red square represents the “Asthma group”, the green triangle represents the “Health group”, and the blue square represents the “QC” (Quality Control group). (**A**) PCA diagram of all samples obtained under POS ions; (**B**) PCA diagram of all samples obtained under NEG ions; (**C**) metabolite database notes.

PCA was performed on 936 metabolites of the healthy and panting groups using SIMCA-P14.1.0 software (Figure 6). The results showed that the two groups of data were significantly separated under the positive or negative ion modes. The biological repeat samples from each group were closely clustered. In the positive ion mode, the first (PC1) and second principal components (PC2) accounted for 16.1% and 8.9% of the total variables, respectively. In negative ion mode, PC1 and PC2 accounted for 18.1% and 5.3% of the total variables, respectively. The higher the PC1 value, the higher the degree of genetic variation among different varieties. The results of the PCA showed significant differences between the healthy and panting groups.

The identified metabolites were annotated using KEGG, HMDB, and LIPIDMAPS. The results showed that 433, 268, and 188 metabolites were enriched in the HMDB, KEGG, and LIPIDMAPS databases, respectively.

#### 3.3.2. Analysis of Differences in Metabolites and Metabolic Pathways Between Samples

**Figure 7:**
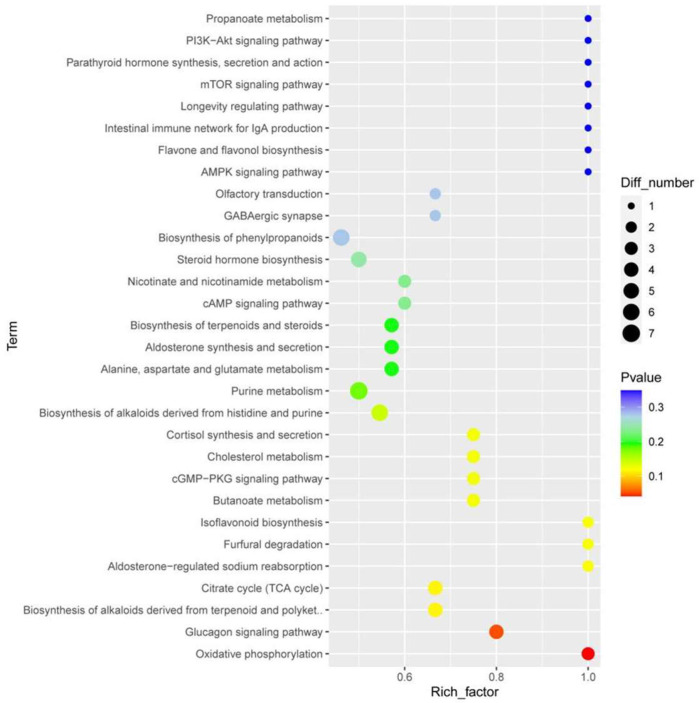
Enrichment analysis of metabolomic data. The horizontal axis represents the enrichment factor, that is, the ratio of the number of differentiated metabolites enriched on a pathway to the background metabolites obtained by sequencing. The ordinate represents the enriched of KEGG functions: the larger the circle, the greater the number of differential metabolites enriched to this function. The color spectrum from blue to red represents uncorrected *p*-values.

To eliminate information that was not relevant to the classification and obtain relevant metabolite information that caused significant differences between the two groups, orthogonal partial least square discriminant analysis (OPLS-DA) was used to filter signals that were not relevant to the model classification. Differentially abundant metabolites (DAMs) between different groups were screened according to a VIP value ≥ 1, *p*-value < 0.05, |log_2_FC| ≥ 1 of PC1of OPLS-DA model. In total, 232 DAMs were detected in the panting and healthy groups, of which 128 were upregulated and 104 were downregulated. We first mapped KEGG, PubChem, and other authoritative metabolite databases through differential metabolites, searched pathway databases, and analyzed the metabolic pathways. The metabolic pathway analysis results for different metabolites between the groups are shown in Figure 7.

The top 10 terms according to *p*-values are oxidative phosphorylation, the glucagon signaling pathway, the TCA cycle, biosynthesis of alkaloids derived from terpenoids and polyketides, furfural degradation, isoflavonoid biosynthesis, aldosterone-regulated sodium reabsorption, butanoate metabolism, cGMP-PKG signaling pathway, and cortisol synthesis and secretion.

### 3.4. Combined Transcriptome and Metabolome Analysis

**Figure 8:**
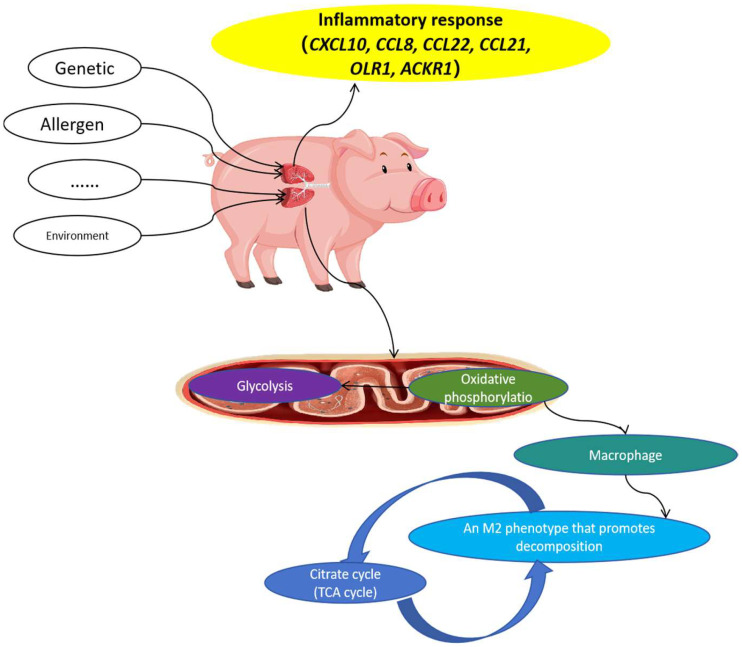
Possible mechanism of asthma formation in Meishan pigs.

As shown in Figure 8, the genes involved in inflammatory responses included *CXCL10*, *CCL8*, *CCL22*, *CCL21*, *OLR1*, and *ACKR1*. Subsequently, the oxidative phosphorylation pathway in lung mitochondria was disrupted, and the succinic acid, riboflavin-5-phosphate, and fumaric acid contents significantly changed compared to those in healthy Meishan pigs. M2 macrophages, which promote decomposition, depend on oxidative phosphorylation in the mitochondria. Disturbance of M2 macrophages ultimately affects the TCA cycle. Specifically, the contents of the metabolites alpha-ketoglutaric acid, alpha-ketoglutaric acid, fumaric acid, and citric acid in the TCA cycle were changed.

### 3.5. Putative Protein Biomarkers for Meishan Pig Asthma

According to the GO pathway results, clustering analysis, and the literature and database searches for each protein, a total of 15 proteins and 7 metabolites were determined to be plausible biomarkers for Meishan pig asthma, which were related to the processes such as, inflammatory response, oxidative phosphorylation, and the citrate cycle (Table 7).

## 4. Discussion

The pathogenesis of asthma is closely related to genetic, neurological, and immune factors, as well as airway inflammation [23,24]. Recently, metabolomics has emerged as a promising tool in multiple fields of biomedicine. In human and animal models, plasma- or serum-based metabolomic studies have elucidated the associations of amino acids, nucleic acid metabolites, and lipid derivatives with asthma and identified the metabolic characteristics of asthma [25]. Serum lipid mediators, hormone concentrations, and blood uric acid levels are associated with asthma severity.

Asthma is a complex disease with multiple etiologies. Metabolomics provides a comprehensive platform to understand the pathophysiological basis of various diseases. As a bridge between the phenotype and genome [26], metabolomics can amplify phenotypic or genomic differences, help identify differences in traits in addition to phenotypic and genetic differences between different germplasms, and improve our understanding of the differences between different germplasms.

In this study, although we have focused on the molecular mechanisms underlying asthma in Meishan pigs through transcriptomics and metabolomics, the role of Mhp infection in the pathogenesis of asthma in these pigs cannot be overlooked.

Mitochondria are the main intracellular consumers of O_2_ and are an important source of oxidative stress [27]. We found that the oxidative phosphorylation pathway in lung mitochondria was disrupted in asthmatic Meishan pigs. Mitochondria produce ROS via electron leakage and molecular oxygen reduction in the respiratory chain [28]. The precursor of ROS is O_2_−, which is produced by mitochondria and NADPH oxidase. Under normal circumstances, 1–3% of the oxygen passing through the mitochondria is used to form ROS. A portion of the energy in all animal cells originates from oxidative phosphorylation in the mitochondria, and the reduction of O_2_ to H_2_O by complexes I and III of the mitochondrial respiratory chain produces intermediate ROS in the mitochondria [29]. Various antioxidants exist in animals, including antioxidant enzymes, such as superoxide dismutase and glutathione peroxidase, and non-enzymatic systems. They can directly remove free oxygen radicals and other oxidative molecules, regenerate damaged biomolecules, and maintain a relative balance between ROS and antioxidant systems. Overproduction of ROS and/or reduced function of the antioxidant defense system leads to cellular oxidative stress, which promotes inflammation and asthma, including airflow obstruction, airway hyperreactivity, and airway remodeling. Our results suggest that the disruption of the oxidative phosphorylation pathway in lung mitochondria may lead to an imbalance between ROS production and antioxidant defense, contributing to the development of asthma in Meishan pigs.

The TCA cycle occurs in the cytoplasm and does not depend on oxygen [30]. We observed significant changes in the contents of metabolites related to the TCA cycle, such as succinic acid, riboflavin—5—phosphate, fumaric acid, alpha-ketoglutaric acid, and citric acid, in asthmatic Meishan pigs compared to healthy ones. Lactic acid is produced under the action of lactate dehydrogenase, with much greater quantities of lactic acid produced in normal lung tissue than in other tissues [31]. These alterations in the TCA cycle may affect energy metabolism and cellular function, further contributing to the pathogenesis of asthma. Previous studies have also shown that metabolite changes in the TCA cycle are associated with asthma severity [32]. Our findings are in line with these reports and suggest that the disturbance of the TCA cycle may play an important role in the development of asthma in Meishan pigs.

Glutathione S-transferases (GSTs) are thought to protect cells from ROS [33]. One of the latest concepts related to asthma to be investigated is the role of GSTs [34]. GSTs are major phase II enzymes involved in metabolic detoxification [35]. Inflammation is key to asthma, and reactive oxygen intermediates that play a role in inflammation are metabolized by GSTs; therefore, abnormalities in GSTs may lead to asthma [36,37]. Although we did not directly measure GST activity in this study, the observed oxidative stress and inflammation in asthmatic Meishan pigs suggest that GSTs may be involved in the pathophysiology of asthma. Future studies could focus on the expression and activity of GSTs in Meishan pigs with asthma to further clarify their role.

Mhp is a significant pathogen that can colonize the respiratory tract of pigs, leading to chronic respiratory diseases. Infection with Mhp can disrupt the normal physiological functions of the respiratory epithelium, which in turn may trigger a series of immune responses and inflammatory cascades. The immune response to Mhp infection is complex and involves both the innate and adaptive immune systems. Arginase 1 (*Arg1*), an enzyme that catalyzes the conversion of arginine to ornithine, is a hallmark of immune-regulating M2 macrophages that produce IL-10 [38]. Our study found that the expression of *Arg1* and related genes may be involved in the immune regulation of asthma in Meishan pigs. During influenza infection in mice, induction of *Arg1* expression is a key feature of lung CD4+ T cells. Conditional ablation of *Arg1* in CD4+ T cells accelerates the virus-specific Th1 response and its resolution, leading to effective viral clearance and reduced lung pathology. Unbiased transcriptomics and metabolomics studies have shown that *Arg1* deficiency, unlike *Arg2* deficiency, leads to alterations in glutamine metabolism [39,40]. Rebalancing this disturbed glutamine flux normalizes the cellular Th1 response. Normal *Arg1* activity allows arginine to produce ornithine, which is essential for cells, thus ensuring optimal glutamine flux into the TCA cycle [41]. In the absence of *Arg1*, the glutamine compensatory reaction reduces TCA activity for ornithine production, thereby affecting the kinetics of the Th1 response [42]. Overall, *Arg1*, which is inherent in CD4+ T cells, can be regarded as a rheostat that regulates the mammalian Th1 life cycle. Recently, Th17 cells, such as CD4+ T cells, have been shown to regulate the involvement of neutrophils in airway inflammation and airway reconstruction in asthma. Our results suggest that Arg1 may play a similar role in the immune regulation of asthma in Meishan pigs, and further studies are needed to elucidate its detailed mechanism.

*RORC* is an upstream regulatory gene and the main effector of Th17 cells [43,44]. *RORC* is involved in asthma pathogenesis via Th17 cells downstream of *RORC*. *RORC* mRNA expression in lung tissue is significantly upregulated in patients with asthma, while IL-17 in the peripheral blood, bronchial lavage fluid, and sputum is significantly increased and positively correlated with airway hyperreactivity [45,46]. In our study, we also observed changes in the expression of *RORC* and related genes in asthmatic Meishan pigs. One study found that *RORC* mRNA levels in peripheral blood mononuclear cells of children with asthma during acute attacks were significantly higher than those in the remitted and normal control groups. *RORC* mRNA expression decreased to normal, whereas the IL-17 level was still significantly higher than that of the normal control group, indicating that *RORC* aggravates airway inflammation and airway hyperreactivity by promoting transcription of the cytokine IL-17 during acute asthma attacks [47]. Previous studies have shown that *RORC* promoter methylation is low in obesity-related asthma. The immune response to allergic asthma is primarily regulated by Th2 lymphocytes, which release IL-4, IL-5, and IL-13. Thus, B cells and eosinophils recruit specific IgE molecules to the respiratory epithelium. RORC is required for naive CD4+ T cells to differentiate into Th17 lymphocytes; *RORC* mRNA expression is higher in obesity-related asthma, while methylation of the *RORC* promoter is lower [48,49]. It is speculated that *RORC* expression is affected by the addition of a methyl group to the carbon of CpG cytosine. Our results are consistent with these previous findings and suggest that *RORC* and Th17 cells may play an important role in the pathogenesis of asthma in Meishan pigs.

Argl is an important enzyme that regulates macrophage function [50] and is a marker of selective macrophage activation. Alveolar macrophages are important sentinels in host lung defense, playing vital roles in maintaining immune regulation, pathogen clearance, and homeostasis [51,52]. M1 macrophages are an important source of many inflammatory cytokines, including TNF-α, IL-1, IL-12, IL-18, and IL-23, which have been identified as important mediators and drivers of chronic inflammatory and autoimmune diseases. The inflammatory response is caused by the aggregation of Th2 lymphocytes, mast cells, eosinophils, and macrophages in the lungs, and is related to the M2 polarization of macrophages [53]. Macrophages are important regulators of allergic asthma and initiators of inflammatory responses associated with lung injury, fibrosis, and goblet cell proliferation [54]. Pulmonary macrophages produce a variety of factors that directly stimulate airway, smooth muscle contractile force and extracellular matrix degradation, and participate in airway pathological remodeling. Analysis of bronchial biopsy specimens revealed an increase in CD206 macrophages in patients with asthma, demonstrating a correlation between the percentage of M2 macrophages and disease severity [55,56,57]. Many circulating M2-like phenotypes have been observed in patients with allergic and bronchial asthma. Additionally, in response to bronchial allergens, macrophages in patients with asthma undergo M2 polarization, thereby supporting Th2-related inflammation. Although airway disease is associated with Th2/M2 inflammation, M1 macrophages may participate in the pathogenesis of asthma by releasing inflammatory cytokines and NO, thereby exacerbating lung injury and airway remodeling. In our study, we found that macrophage polarization and function may be altered in asthmatic Meishan pigs, which is consistent with previous reports. Future studies could further investigate the role of macrophages in the development and progression of asthma in Meishan pigs.

The Notch pathway plays an important role in the occurrence and development of asthma. The Notch pathway affects lung tissue development, determines the direction of cell differentiation, and regulates the development of alveoli and pulmonary blood vessels [58]. The Notch pathway, which is known to be involved in cell differentiation and immune regulation, may also be modulated by Mhp infection. Mhp-induced cytokines and growth factors could potentially affect the expression and activation of Notch receptors and ligands, thereby altering the Notch signaling pathway. The Notch pathway is involved in T-cell regulation, which affects Th17 cells, Tregs, dendritic cell expression, and other pathways by altering the Th1/Th2 balance, leading to the occurrence and development of asthma [59]. The Notch pathway also participates in the pathological changes in airway remodeling in asthma by altering the infiltration of various inflammatory cells, such as lymphocytes and eosinophils, promoting the metaplasia of airway goblet cells and airway mucus secretion [60]. Notch signaling molecules are dynamically expressed during lung development and may play a key role in regulating the differentiation and development of the alveolar epithelium and vascular endothelial cells. Some studies have reported that the application of gamma-secretase inhibitor DAPT to inhibit the Notch signaling pathway can inhibit angiotensin II-induced pulmonary vascular remodeling and reduce pulmonary artery pressure [61,62,63], providing a new idea for the treatment of pulmonary hypertension. However, the regulatory mechanisms of the Notch pathway have not been fully elucidated and more in-depth studies are needed. Our results suggest that the Notch pathway may be involved in the pathogenesis of asthma in Meishan pigs, and further studies are warranted to explore its detailed mechanism and potential therapeutic targets. However, the value of its application in clinical treatment requires further exploration.

YPEL family proteins are located in centrosomes near the interphase nucleolus and mitotic organs during mitosis [64]. Based on their subcellular localization, YPEL4 may play an important role in the cell cycle and proliferation. YPEL4 may also mediate adrenal cell proliferation by regulating the mitogen-activated protein kinase signaling pathway [65]. This molecular mechanism may also play an important role in lung diseases. Although the function of YPEL4 is largely unknown, further research may confirm its functional importance and the underlying molecular processes in the lungs and other diseases, which would make YPEL4 a therapeutic target. In our study, we observed changes in the expression of *YPEL4* and related genes in asthmatic Meishan pigs, suggesting that *YPEL4* may be involved in the cell proliferation and airway remodeling processes in asthma. Future studies could focus on elucidating the exact role of YPEL4 in the pathogenesis of asthma.

Asthma is characterized by an increased proliferation of smooth muscle cells in the airway walls [66]. Hasaneen et al. studied the dual ERK and phosphatidylinositol 3-kinase (PI3K) pathways and their regulation of airway smooth muscle cell proliferation in asthmatics [67]. In non-asthmatic cells, growth is controlled by mitogens, which pass through dual signaling pathways, namely the ERK-and PI3K-dependent pathways [68]. In asthmatic cells, the PI3K pathway is dominant because of the upregulation of the endogenous MAPK inhibitor MAPK phosphatase-I. This inhibitor restricts the ERK signaling pathway in asthmatic cells under mitotic stimulation, making the PI3K pathway dominant. Ultimately, this study suggests that PI3K is an important target for smooth muscle hyperplasia in asthma. Notably, the PI3K signaling pathway plays an important role in the dual pathway between the non-asthmatic PI3K and MAPK signaling pathways. Naturally, the major vault protein (MVP) inhibits YPEL4’s ability to activate the transcription factor Elk-1 in the MAPK signaling pathway. In terms of interactions with YPEL4, specific inhibitors of MVP may allow the MAPK pathway to work alongside the PI3K pathway simultaneously, suggesting that specific inhibitors of MVP may serve as therapeutic targets for asthma by promoting the activity of YPEL4 [69]. By interacting with MVP, YPEL4 participates in the activities and functions of Elk-1. Thus, YPEL4 plays an important role in regulating the MAPK transduction pathway. Studies have shown that YPEL4 mediates cell cycle progression and cell proliferation. The function of YPEL4 is not fully understood; therefore, further research may contribute to the understanding of YPEL4 as a potential therapeutic target.

The expression of *RAPGEF3*, also known as *EPAC*, plays a role in neutrophil dysfunction and airway smooth muscle remodeling [70]. *RAPGEF3* may work synergistically with another candidate gene, cadherin-6 (*CDH6*), to influence asthma risk [71]. Both genes have been found to be involved in cell–cell connectivity, which may play a key role in the pathophysiology of asthma. In our study, we also detected changes in the expression of *RAPGEF3* and related genes in asthmatic Meishan pigs, indicating that *RAPGEF3* may be involved in the airway smooth muscle remodeling process in asthma. Future studies could investigate the interaction between *RAPGEF3* and other genes in the context of asthma pathogenesis.

Airway mucus hypersecretion is an important pathophysiological change in asthma and the main cause of death in severe asthma [72,73], but the mechanism underlying the regulation of mucus hypersecretion in asthma is still unclear. Mucins are the main component of airway mucus and play important roles in viscoelasticity [74]. A recent study found that myristoylated alanine-rich C kinase substrate (MARCKS) is a key molecule in mucus secretion [75]. The secretory inductor PKC phosphorylates MARCKS, which is transferred from the serous membrane to the cell membrane, where MARCKS dephosphorylates, binds to actin and myosin and interacts with the mucous granular membrane [76]. Through this mechanism, the mucins attach to the contractile cytoskeleton, migrate to the cell periphery, and undergo exocytosis. MARCKS regulates the extracellular secretion of mast cell particles in a PKC-dependent manner by regulating the availability of membrane inosine phosphate, which is required for particle fusion with the plasma membrane [77]. In our study, we found that the expression of *MARCKS* and related genes may be involved in the regulation of airway mucus secretion in asthmatic Meishan pigs. Future studies could further explore the role of *MARCKS* in the pathophysiology of asthma and its potential as a therapeutic target.

## 5. Conclusions

In this study, through a combined multi-omics analysis of asthma in Meishan pigs, the complex molecular pathogenesis has been systematically revealed. During the occurrence of asthma, the abnormal expression of inflammation-related genes such as *CXCL10* and *CCL8* is closely intertwined with the disorders of mitochondrial oxidative phosphorylation and the tricarboxylic acid cycle, constituting a complex regulatory network. The metabolomics data further confirm the crucial roles of key metabolites such as succinic acid and riboflavin-5-phosphate in the asthma process. These findings not only fill the gaps in the research field of asthma in Meishan pigs, providing important bases for the precise treatment and resistance breeding of asthma in this pig breed, but also offer a unique perspective for an in-depth understanding of the general mechanisms underlying asthma pathogenesis.

In summary, this study provides valuable references for the veterinary field and potentially for human asthma research. In the future, based on these achievements, we will further validate the causal relationships of key genes and metabolites, expand the application of research findings in cross-species asthma research, and strive to provide more effective strategies for the prevention and treatment of asthma.

## Figures and Tables

**Table 1:** Primer design for six genes.

Gene Name	Primer Name	Primer Sequence
*VMP1*	17668F	CAGGCTAAGGGTCCAGTTGG
	17668R	GTAACACGTGGAGTTCCCGT
*ATP5IF1*	27072F	AGGGGTCTAATCGGAGCTGT
	27072R	TTAGGAGTTCCCGTTGTGGC
*ZNF205*	29375F	TGGCTCGGTGGAAAGGAATC
	29375R	GAGGCATATGGAGGTTCCCG
*FOXS1*	33613F	GGCTCATGGACAGTGACACA
	33613R	GCTGAGGCTCAGTTTCCCAT
*LGALS3BP*	36383F	GCCACCTCAGAGCAACAGAT
	36383R	GCCCTGGGAAGCCAACTATT
*18S*	18S-F	AGTCGCCGTGCCTACCAT
	18S-R	CGGGTCGGGAGTGGGTAAT

**Table 2:** Reaction conditions of the genomic DNA removal reaction.

Reagent	Dosage
5 × gDNA Eraser Buffer	2.0 μL
gDNA Eraser	1.0 μL
Total RNA	1 μg
RNase Free dH_2_O	up to 10 μL

**Table 3:** Reagent and dosage required for reverse transcription reaction.

Reagent	Dosage
5 × PrimeScript^®^ Buffer 2	4.0 μL
PrimeScript^®^ RT Enzyme Mix I	1.0 μL
RT Primer Mix	1.0 μL
Reaction liquid	10.0 μL
RNase Free dH_2_O	up to 20 μL

**Table 4:** Reagents and dosages required for qPCR reaction system.

Reagent	Dosage
qPCR Mix	15 μL
Forward Primer (10 μM)	0.5 μL
Reverse Primer (10 μM)	0.5 μL
Template DNA	2 μL
ddH2O	30 μL

**Table 5:** Various parameters required for qPCR reaction.

Gene Name	HOLD		CYCLE			
*VMP1*	95 °C 3 min	1 cycle	95 °C 15 s	62 °C 20 s	72 °C 20 s	40 cycle
*ATP5IF1*	95 °C 3 min	1 cycle	95 °C 15 s	57 °C 20 s	72 °C 20 s	40 cycle
*ZNF205*	95 °C 3 min	1 cycle	95 °C 15 s	57 °C 20 s	72 °C 20 s	40 cycle
*FOXS1*	95 °C 3 min	1 cycle	95 °C 15 s	57 °C 20 s	72 °C 20 s	40 cycle
*LGALS3BP*	95 °C 3 min	1 cycle	95 °C 15 s	57 °C 20 s	72 °C 20 s	40 cycle
*18S*	95 °C 3 min	1 cycle	95 °C 15 s	57 °C 20 s	72 °C 20 s	40 cycle

**Table 6:** Basic data before and after quality control.

#Sample_ID	Raw_Reads	Clean_Reads	GC%	Mapped_Reads	Mapped_Rate (%)	Raw_Q30%	Clean_Q30%
TWL1	111,283,926	109,896,962	56.7	105,336,238	95.85	95.15	95.57
TWL2	109,933,392	108,522,062	57.62	104,626,119	96.41	95.54	95.93
TWL3	124,906,660	123,326,384	56.82	118,553,652	96.13	95.3	95.69
TWL4	120,439,624	118,930,184	56.71	113,138,284	95.13	95.55	95.94
TWL7	143,298,942	141,539,198	55.96	133,995,158	94.67	95.42	95.82
TWL9	123,154,462	121,569,990	56.17	115,758,944	95.22	95.14	95.54
TWL10	259,626,684	256,110,084	56.12	241,742,308	94.39	95.52	95.93
TWL11	124,657,442	122,835,734	56.8	117,381,827	95.56	95.45	95.85
TWL12	116,285,756	114,677,324	56.88	109,149,876	95.18	95.61	95.98
TWL14	137,001,130	135,553,352	56.03	128,301,247	94.65	95.59	95.96
TWL15	118,516,262	117,123,334	56.07	111,759,085	95.42	95.61	96.01
TWL17	127,660,270	126,189,658	53.5	114,264,735	90.55	95.79	96.19
TWL18	125,615,062	124,107,130	53.79	113,905,523	91.78	92.46	92.83
TWL19	124,255,398	122,398,050	54.96	112,373,649	91.81	96.11	96.45
TWL20	115,767,154	114,226,326	53.97	105,727,887	92.56	95.73	96.14
TWL21	123,344,164	121,911,038	54.69	114,828,006	94.19	95.24	95.65
TWL23	131,278,054	129,629,036	54.19	120,140,190	92.68	95.65	96.06
TWL24	119,705,652	118,399,398	54.57	110,407,438	93.25	95.74	96.13
TWL25	132,343,992	130,915,808	54.32	121,476,778	92.79	95.08	95.45
TWL27	109,264,138	108,004,800	54.14	99,278,012	91.92	95.77	96.16
TWL28	117,222,000	115,854,562	53.43	106,006,924	91.5	95.51	95.94
TWL29	115,545,422	114,174,216	55.51	106,524,543	93.3	95.84	96.21

Sample_ID is the name of the sample. Raw_Reads are the statistical raw sequence data. Clean_Reads are data filtered under certain conditions based on the raw reads. GC% represents the GC content of the entire sequence. Owing to the high GC preference of second-generation sequencing, the higher the depth, the higher the GC content. Mapped_Reads indicates the number of reads matched to the reference genome. Mapped_Rate represents the ratio of the number of reads compared to the reference genome. Q30% represents the percentage of total bases with Phred values > 30.

**Table 7:** Putative protein biomarkers for Meishan pig asthma.

Functional Class	Gene Symbol or Metabolite Name
Inflammatory response	*CXCL10*, *CCL8*, *CCL22*, *CCL21*, *OLR1*, *ACKR1*
Oxidative phosphorylation	Succinic acid, riboflavin-5-phosphate, fumaric acid
Citrate cycle (TCA cycle)	alpha-ketoglutaric acid; succinic acid; fumaric acid; Citric acid
Others	*Arg1*, *RORC*, *IL17A*, *TNFA*, *Notch3*, *YPEL*, *YPEL4*, *RAPGEF3*, *MARCKS*

## Data Availability

The original contributions presented in the study are included in the article; further inquiries can be directed to the corresponding author.
